# Prevalence of Bronchiectasis in Asthma according to Oral Steroid Requirement: Influence of Immunoglobulin Levels

**DOI:** 10.1155/2013/109219

**Published:** 2013-11-13

**Authors:** Manel Luján, Xavier Gallardo, María José Amengual, Montserrat Bosque, Rosa M. Mirapeix, Christian Domingo

**Affiliations:** ^1^Servei de Pneumologia, Hospital de Sabadell, Corporació Sanitària i Universitària Parc Taulí, Parc Taulí 1, Sabadell, 08208 Barcelona, Spain; ^2^Departament de Medicina, Universitat Autònoma de Barcelona (UAB), Campus de la UAB, Cerdanyola del Vallès, 08193 Barcelona, Spain; ^3^Ciber de Enfermedades Respiratorias (CIBERES), Carretera Soller Km 12, Illes Balears, 07110 Bunyola, Spain; ^4^Servei de Diagnòstic per la Imatge, UDIAT, Corporació Sanitària i Universitària Parc Taulí, Parc Taulí 1, Sabadell, 08208 Barcelona, Spain; ^5^Servei de Laboratori, UDIAT, Corporació Sanitària i Universitària Parc Taulí, Parc Taulí 1, Sabadell, 08208 Barcelona, Spain; ^6^Servei de Pediatria, Unitat de Pneumologia, Al.lèrgia i Fibrosi Quística, UDIAT, Corporació Sanitària i Universitària Parc Taulí, Parc Taulí 1, Sabadell, 08208 Barcelona, Spain; ^7^Departament de Ciències Morfològiques, Universitat Autònoma de Barcelona (UAB), Campus de la UAB, Cerdanyola del Vallès, 08193 Barcelona, Spain

## Abstract

*Purpose*. To establish the prevalence of bronchiectasis in asthma in relation to patients' oral corticosteroid requirements and to explore whether the increased risk is due to blood immunoglobulin (Ig) concentration. *Methods*. Case-control cross-sectional study, including 100 sex- and age-matched patients, 50 with non-steroid-dependent asthma (NSDA) and 50 with steroid-dependent asthma (SDA). Study protocol: (a) measurement of Ig and gG subclass concentration; (b) forced spirometry; and (c) high-resolution thoracic computed tomography. When bronchiectasis was detected, a specific etiological protocol was applied to establish its etiology. *Results*. The overall prevalence of bronchiectasis was 12/50 in the SDA group and 6/50 in the NSDA group (*p* = ns). The etiology was documented in six patients (four NSDA and two SDA). After excluding these patients, the prevalence of bronchiectasis was 20% (10/50) in the SDA group and 2/50 (4%) in the NSDA group (*P* < 0.05). Patients with asthma-associated bronchiectasis presented lower FEV_1_ values than patients without bronchiectasis, but the levels of Ig and subclasses of IgG did not present differences. *Conclusions*. Steroid-dependent asthma seems to be associated with a greater risk of developing bronchiectasis than non-steroid-dependent asthma. This is probably due to the disease itself rather than to other influencing factors such as immunoglobulin levels.

## 1. Introduction

Bronchiectasis is defined as abnormal, irreversible thick-walled dilatation of the bronchi and represents the end stage of a variety of pathological processes. Caused by the inflammatory reaction of the bronchi and their frequent chronic bacterial colonization, bronchiectasis usually presents with recurrent lower respiratory tract infections and chronic mucopurulent sputum production.

Bronchiectasis often goes unrecognized, even when characteristic features are present and appropriate diagnostic techniques are readily available. In a recent study the etiology of the condition was identified in only 57% of patients [1]. The etiological spectrum of the disease has changed notably since it was first described. In the preantibiotic era, bronchiectasis was almost exclusively due to untreated or suboptimally treated lung infections, including pneumonia or tuberculosis [2]. Today, although the majority of identified causes of bronchiectasis are infections (24% [2, 3] and 29% [4]), associations have been reported with new entities like adulthood cystic fibrosis [5, 6], humoral immunodeficiency including common variable immunodeficiency [7, 8], and systemic diseases [9]. On the other hand, despite the improvements in the control of pulmonary infectious diseases, the incidence of bronchiectasis does not seem to be declining—perhaps because the widespread use of high-resolution chest tomography (HRCT) has allowed easy diagnosis of its clinically silent minor forms. The use of CT to diagnose bronchiectasis has been validated in various studies [10]. Overall, today, the etiology is identified in fewer than 50% of patients with the condition [4, 11].

Several studies have attempted to establish an association between asthma and bronchiectasis [12–14]. Recently, in a study of 1680 patients with asthma, around 3% had radiographic bronchiectasis, and around 50% of patients with bronchiectasis had severe asthma. Unfortunately, the authors did not rule out other causes of bronchiectasis [15]. In this connection, a role for immunoglobulin (Ig) and IgG subclass deficiency, classically associated with the development of bronchiectasis [7, 8], has been suggested in certain cases of bronchial asthma [16, 17]. 

 The aim of the present study was to compare the prevalence of bronchiectasis in a cohort of patients with severe steroid-dependent asthma (SDA) and in another with non-steroid-dependent asthma (NSDA), in order to establish whether the SDA group has an increased associated risk of developing bronchiectasis. We also determined the Ig and IgG subclass blood levels in an attempt to correlate them with the development of bronchiectasis in either group.

## 2. Material and Methods

 The study was performed between 2004 and 2011 at the Corporació Sanitària Universitària Parc Taulí, a 760-bed university hospital with a reference population of 400,000 inhabitants. The study design was a single-center, prospective case-control study. Each patient in the SDA group was matched for age and sex status with one in the NSDA group. Patients were consecutively recruited from our institution's asthma out-patient clinic [18] and complete patient evaluation was performed prospectively and lasted less than three months per patient.

### 2.1. Patients

#### 2.1.1. Inclusion Criteria

 All patients were nonsmokers and were treated in accordance with the severity of their disease following international guidelines [19].

#### 2.1.2. NSDA (GINA Steps  I to  IV)

 The maximum treatment received by NSDA patients was salmeterol 50 *μ*g bid, fluticasone 500 *μ*g bid, salbutamol as rescue medication, and oral antileukotrienes. None had received oral steroids during the year prior to inclusion in the study.

#### 2.1.3. SDA (GINA Step  V)

 In addition to the treatment just described, SDA patients were also given oral steroids at least 7.5 mg/day of oral prednisone (or equivalent). No patient was receiving immunomodulatory treatment [20–22] since in accordance with our policy these treatments are not prescribed to our patients until corticodependence is verified [23].

A stabilization period of three months was required before admission, during which the FEV_1_ did not change more than 5% and patients did not report any deterioration in their clinical symptoms. Failure in full-tapering of corticosteroids for at least three months was also required.

### 2.2. Study Protocol

A general protocol was applied to the entire study population (general instrumentation) to establish the prevalence of bronchiectasis and, in a second step, a specific etiologic protocol to determinate the cause(s) of bronchiectasis in patients in which this entity was documented (specific instrumentation).

#### 2.2.1. General Instrumentation

The following tests were performed in all patients. (a) Blood analysis was performed including red and white blood cell count, platelet count, renal, hepatic biochemical parameters, rheumatoid factor, autoantibody screening test and determination of IgG, IgM, and IgA by nephelometry, and IgE. IgG subclass levels were determined by radiated immunodiffusion. (b) Forced spirometry with bronchodilator test was performed in accordance with local guidelines [24] (MedGraphics system 1070 Series 2E/1085). The bronchodilator test was considered positive when the FEV1 increased by 12% and 200 mL. Reference values for the Mediterranean population were used [25], and normal spirometry was defined as FEV1 ≥ 80% of predicted value and FEV1/FVC ≥ 0.7. (c) Multidetector CT scanner (Sensation 16; Siemens, Erlangen, Germany) that acquires thin-slice images of the whole chest, with 0.5–1 mm thick slices, during a single breathhold was performed. These scanners produce true isotropic voxels, allowing image reconstructions in which the *Z* dimension (slice thickness) is the same dimension as the *X* and *Y* (in plane) resolution. A radiologist skilled in HRCT and blind to the study interpreted the scans, classifying patients as having or not bronchiectasis and assessing the severity of the bronchiectasis in accordance with Reiff et al.'s classification [26]. Following this score, each lung was evaluated according to individual lobes, with the lingula being considered a separate lobe. Each lobe was scored for extent of involvement: 0 = none, 1 = involvement of one or a partial segment, 2 = involvement of two or more segments, and (d) Skin prick test was performed.

#### 2.2.2. Specific Instrumentation

This part of the protocol was only performed in patients who had shown radiological evidence of bronchiectasis ruling out other causes of the disease: (a) clinical assessment to detect chronic sputum production and remote infections in childhood or prior to the onset of asthma symptoms; (b) blood analysis: alpha-1-antitrypsin deficiency, precipitins for aspergillus, and genetic profile of cystic fibrosis; (c) sweat test; (d) nasal potential difference; (e) saccharin test; and (f) seminogram in males.

#### 2.2.3. Classification of Patients according to the Specific Protocol

In patients with a diagnosis other than asthma, the bronchiectasis was attributed to this alternative diagnosis. These patients were classified as having non-asthma-related bronchiectasis and were excluded from the study. Bronchiectasis in patients without alternative etiological diagnosis was considered asthma to be related to. The alternative diagnostics were defined as follows: (a) alpha-1-antitrypsin deficiency was diagnosed if serum levels were below 80 m/dL; (b) common variable immunodeficiency: presence of IgG or subclass deficiency (below 700 mg/dL for total IgG, and 550, 130, 21 and 20 mg/dL for IgG1 to IgG4 subclasses, resp.) with IgA or IgM levels below normal lower limit (below 70 and 40 mg/dL, resp.); (c) adulthood cystic fibrosis: presence of a mutation associated in the literature with cystic fibrosis with altered sweat test; (d) bronchopulmonary allergic aspergillosis: central bronchiectasis plus positive precipitins for aspergillus; (e) postinfectious bronchiectasis: medical history of documented pulmonary infection (tuberculosis or not) in the site of bronchiectasis. 

### 2.3. Statistical Methods

#### 2.3.1. Sample Size Calculation

Since the only reliable data on the prevalence of bronchiectasis in steroid-dependent asthmatic patients were reported in our earlier pilot study [27], we calculated the sample size from our preliminary results. We also assumed that the prevalence in the non-steroid-dependent group would be very low (probably similar to the general population) and calculated the sample size assuming a prevalence of 20% in the SDA group and 4% in the NSDA group, an alpha risk of 0.05 and a beta risk of 0.20. In a unilateral contrast, we needed 48 patients in each branch. We estimated a loss rate <0.05 and therefore included 100 patients divided into two groups of 50 according to their oral steroid requirements. An interim analysis was not planned. For the sample size calculation we used the Gramno program (IMIM-Barcelona).

#### 2.3.2. Data Entry and Analysis

Statistical Package for the Social Sciences (SPSS version 19.0) was used for data entry and analysis. The results are given as mean (SD) values. Statistical comparisons between groups were performed using the two-tailed unpaired Student's *t* test for continuous variables. The Chi-squared test was used to compare proportions between the two groups. To detect the risk of developing the disease a stratified analysis (Mantel and Hanszel method) was used and a stepwise regression logistic analysis was performed later to detect independent factors for developing bronchiectasis; the model included all variables with *P* < 0.1 in the univariate analysis. 

The protocol was approved by the Ethics Committee of our Institution, and informed consent was obtained from each participant.

## 3. Results

Fifty patients were included in the SDA group and 50 age- and sex-matched controls in the NSDA. None of them had a history of tobacco exposure. No differences in age (56.9 ± 13.1 for controls and 57.8 ± 10.8 for cases, *p* = ns) or sex (M/F 15/35 for each group) were found. Time from clinical diagnosis to inclusion in the study was 23.7 ± 14.5 years in the SDA group and 16.02 ± 11.4 years in the NSDA group (*P* < 0.05). The mean daily steroid dose in SDA group was 14.9 ± 8 mg of methylprednisolone/day or equivalent.

### 3.1. Spirometry and Immunology

 As stated in Table 1, the SDA group presented more severe obstruction than the NSDA group. Moreover, 38% of patients (19/50) presented normal spirometric values in the NSDA group and only 4/50 (8%) in the SDA group (*P* < 0.01).

#### 3.1.1. Immunology

Mean values of IgA, IgG, and IgG subclasses 1, 2, and 3 were lower in the SDA group. We did not detect combined deficiency of IgG (or subclasses) with low IgA or IgM values corresponding to a common variable immunodeficiency (Table 1). 

#### 3.1.2. Diagnosis of Bronchiectasis

Twelve out of 50 patients in the SDA group were diagnosed with bronchiectasis by the HRCT scan. Severity of the condition according to the classification of Reiff et al. [26] was 4.17 ± 3.29 points. In the NSDA group, six patients had bronchiectasis (*p* = ns in comparison with SDA group), with a score of 3.17 ± 1.16 on the classification of Reiff (*p* = ns).

Applying the etiology study protocol, an alternative diagnostic etiology was found in six cases: two of them in the SDA group (both adult cystic fibrosis) and four in the NSDA group (one tuberculosis, one bronchiectasis postpneumonia, and two cases of adult cystic fibrosis). After ruling out those cases with a definitive etiology of bronchiectasis, the prevalence of asthma-associated bronchiectasis was 10/50 (20%) in the SDA group and 2/50 (4%) in the NSDA group (*P* < 0.05). 11/12 (91%) of patients with asthma-associated bronchiectasis were documented in patients with abnormal spirometry (10 in the SDA group and 1 in the NSDA group, *P* < 0.05).

Table 2 compares the patients with asthma-related bronchiectasis (*n* = 12) and those patients without (*n* = 88). Patients with asthma-associated bronchiectasis were older, with longer-term asthma history, and had lower FEV_1_ and FEV_1_/FVC values. Conversely, the IgG, IgA, and IgG subclasses mean values did not differ between patients with and without asthma-associated bronchiectasis. Only IgM mean values were higher in the patients with bronchiectasis. Using normality cut-off points, 7 patients in the nonbronchiectasis group and 2 in the bronchiectasis group presented isolated low values of IgG (*p* = ns), and only 5 patients with isolated IgA deficiency were found (4 in nonbronchiectasis and 1 in bronchiectasis, *p* = ns). Moreover, comparing patients in the SDA group with asthma-related bronchiectasis (*n* = 10) with the remaining forty patients without, neither the daily steroid dose nor the levels of immunoglobulins and IgG subclasses (with the exception of IgM values) were associated with the presence of bronchiectasis (data not shown).

Finally, after adjusting for immunoglobulin levels, a stepwise regression logistic analysis showed that only age (aOR 1.08, 95% CI: 1.01–1.16) and steroid dependence (aOR 6.9, 95% CI: 1.35–35.09) were associated with the presence of asthma-related bronchiectasis. 

## 4. Discussion 

 To establish the prevalence of a pathology, a preliminary appraisal using a cross-sectional study is required. Patient selection and sample size estimation must be performed with care. Given the lack of reliable data in the literature, we based our calculations of the sample size on our earlier pilot study [27]. To control the confounding variables and to increase the efficiency of the study, each patient with steroid-dependent asthma was matched with a patient from the non-steroid-dependent group of the same race, sex, and age range to ensure the comparability of the groups. There were no differences in terms of age or sex. Obviously, the degree of bronchial obstruction differed: SDA patients presented moderate/severe obstruction while in NSDA patients FEV_1_ values were almost normal. 

 From the point of view of the methodology used to diagnose bronchiectasis and its causes, our study presents two innovative features. First, we established the diagnosis using a HRCT scan in stable patients. The original CT scans designed to assess airway structure involved thin-slice images (typically 1-2 mm axial), which were acquired using a “stop and shoot” protocol and were reconstructed using an edge-enhancing algorithm. Conversely, the high-resolution CT (HRCT) protocol allows the measurement of the airways in true cross section at any location, using retrospective reconstruction of the images to achieve a cross-sectional image of the airway [28].

Second, after confirming the diagnosis, we performed an etiological diagnosis of bronchiectasis applying our pneumology service's protocol which includes innovative techniques for screening infrequent illnesses [29, 30] such as the determination of nasal potential difference [31], an examination now accepted worldwide as a diagnostic criterion for cystic fibrosis. So the comparison of proportions was carried out among patients with bronchiectasis correctly diagnosed by HRCT scan in whom other etiological causes of bronchiectasis had been ruled out. 

 To evaluate bronchiectasis severity, we used the easily reproducible CT grading system described by Reiff and coworkers [26]. The finding of at least two lobes and a minimum of three segments affected in bronchiectasis patients supports the idea that asthma causes a generalized inflammation of the airways. As can be seen, though SDA patients had higher scores, no statistical differences were found between groups.

 The literature review takes us back to the 1960s, when Dunnill's autopsy findings recorded the presence of bronchiectasis in 4/20 (25%) asthmatic subjects [12]. For many years Dunnill's results were regarded as unreliable since they were obtained from necropsies of patients who died in status asthmaticus, a situation that increases the presence of mucous plugs which disrupt and dilate the airway wall. Years later, authors studying lung parenchyma of asthmatic patients using HRCT found that asthmatics present more radiological abnormalities than normal subjects, such as bronchial dilatation and even bronchiectasis, probably related to permanent airway remodeling. 

 Though some of these studies used HRCT scan, the method and/or the moment used for accepting the diagnosis of bronchiectasis were often inadequate, or no etiological differential diagnosis of bronchiectasis was performed. Consequently a wide range of prevalence results have been found. Paganin et al. [13] reported a prevalence of cylindric bronchiectasis of up to 80% in severe nonallergic asthmatics. Similarly, Lynch et al. [14] found bronchial dilatation in 77% of compensated asthmatic subjects. Since these findings have not been considered reliable, recent investigations [11] of the causative factors of bronchiectasis have excluded bronchial asthma from the disease's etiological spectrum. 

 In a recent study, Anwar et al. [1] found asthma to be the aetiology of bronchiectasis in 3% of the cases. Oguzulgen et al. [15] found that asthmatics with bronchiectasis mostly had severe persistent asthma (49.0%), while pure asthmatics mostly had mild persistent and intermittent asthma (69.4%). More recently, in a retrospective data based study, Bisaccioni et al. [32] found that among the 245 asthma patients evaluated in whom CT scans of the chest were taken for 105 patients, 26 (24.8%) showed bronchiectasis. Only 2.5% of their cohort was taking oral steroids, but 98.7% had an abnormal lung function and 94.6% were considered uncontrolled. Thus, many of these patients (probably the majority) were probably SDA patients who at that moment were not receiving oral steroids. Thus, our study is the first to establish a reliable prevalence of bronchiectasis in both stabilized SDA (18%) and NSDA (4%) obtained after ruling out causes other than asthma. The prevalence of bronchiectasis in SDA we found is thus probably in concordance with Bisaccioni's results [32]. Furthermore, using a comparison of proportions, we were able to establish the increased risk of developing bronchiectasis in the SDA group compared with the NSDA group. 

 Regarding the PFT results, we observed that the degree of airway obstruction was more severe in SDA than in NSDA. Asthma and bronchiectasis coexist in many patients, and it has been shown that bronchiectasis can contribute to severe and difficult-to-control asthma with pulmonary complications [33]. In this population, PFTs are frequently abnormal [32]. Therefore, our results are as expected.

 In patients with asthma, especially in those with severe disease, several studies have reported a high incidence of Ig and IgG subclass deficiency and suggest that this may play a role in the pathogenesis of the disease [16, 17]. We observed that the level of immunoglobulins was lower in the SDA group, lower even than the normal values found in our laboratory. This may be because the steroids themselves lower the IgG_1_ values (the most abundant fraction of the IgG subclasses) and thus the total IgG value as well. However, comparing the immunoglobulin values in patients with asthma-related bronchiectasis with those without bronchiectasis there were no significant differences; the only relevant finding was the higher IgM value in patients with bronchiectasis, possibly because they had presented infection more recently. Therefore, Ig values do not appear to play an important role in the pathogenesis of bronchiectasis in asthmatic patients.

 The prevalence of bronchiectasis is statistically higher in SDA than in NSDA. Steroid dependency is associated with a higher risk of developing bronchiectasis, and the development of the condition is not due to the circulating levels of immunoglobulins. So it seems logical to attribute this increased risk to the evolution of steroid-dependent asthma, with higher levels of inflammation and bronchial remodeling, higher levels of formation of thick, difficult-to-clear mucous plugs, and progressive obstruction of the airway with a low degree of reversibility. 

 The study has several limitations. The first is the fact that we do not know the prevalence of bronchiectasis in the general nonsmoking population and in NSDA. For this reason we assumed a high prevalence (4%), to be sure that the calculation of the sample size was sufficient. The second is the selection bias in patient recruitment. The SDA patients were recruited from the study of difficult-to-control asthma carried out at our hospital [18] and the NSDA patients from the pneumology service at our institution. In the SDA group there is no chance of a selection bias, but in the NSDA it may be that the patients assessed, though not steroid dependent, presented higher severity than other apparently equally severe cases treated in primary care. If so, the prevalence of bronchiectasis obtained in this group would be greater than the real figure. However, this would not affect the significance of our results, and the value obtained for the increased risk of developing bronchiectasis in the SDA group would still be valid. In addition, asthma symptoms had lasted longer in the SDA group than in NSDA. But although the duration of the disease differs in the two groups, we stress that in the NSDA group the mean duration period was also very long (16 years), which may have given these patients the opportunity to develop bronchiectasis. Finally, gastroesophageal reflux (GER) may be present in patients with asthma [32]. Although rare, GER can be the causative factor of bronchial aspirations. No special testing to rule out GER was performed, but, when present, bronchial aspirations are not usually the causative agent of bronchiectasis. Thus we do not believe that this entity played an important role in our cohort of patients. 

Therefore, after having ruled out other potential causative factors, we believe that our results show a clear association between SDA and bronchiectasis that is related not to the immunological situation of the patient but probably to the disease itself. Future studies should explore whether the risk of infection and colonization by germs that are more frequent in patients with bronchiectasis than in patients with asthma is higher in SDA patients.

## Figures and Tables

**Table 1:** Spirometric and Ig/IgG subclass plasmatic values in SDA and NSDA patients.

		SDA	NSDA	*P*
Forced spirometry	FEV1 L (%)	1.52 ± 0.53 (52.1 ± 18.6)	2.02 ± 0.81 (78.13 ± 21.09)	0.01 0.01
	FVC L (%)	2.70 ± 0.67 (82.62 ± 15.96)	2.92 ± 1.06 (84 ± 15.97)	Ns
	FEV1/FVC %	56.27 ± 12.47	69.38 ± 13.16	<0.01

Ig (mg/dL)	IgA (*N* = 70–400)	207 ± 114	255 ± 105	0.03
	IgM (40–230)	152 ± 137	113 ± 56	ns
	IgG (700–1600)	784 ± 226	1032 ± 318	<0.001

IgG subclass (mg/dL)	IgG1 (*N* = 550–1270)	485 ± 177	586 ± 241	0.01
	IgG2 (*N* = 130–700)	267 ± 108	334 ± 144	0.01
	IgG3 (*N* = 21–180)	58 ± 28	87 ± 78	0.01
	IgG4 (*N* = 20–150)	23 ± 16	29 ± 33	ns

**Table 2:** Comparison of demographic conditions, spirometric values, and plasmatic values of  Ig and IgG subclass in patients with and without asthma-associated bronchiectasis.

		Asthma-associated bronchiectasis (*n* = 12)	Non-asthma-associated bronchiectasis (*n* = 88)	*P* value
Baseline conditions	Age	64.5 ± 8	56.4 ± 12.4	<0.05
	Time from diagnosis (yr)	30.14 ± 7.2	16.98 ± 12.4	<0.05

Spirometric values	FEV1 (L)	1.31 ± 0.51	1.82 ± 0.73	<0.05
	FEV1 %	61.9 ± 29.9	70.9 ± 19.8	ns
	FVC (L)	2.51 ± 0.65	2.85 ± 0.91	ns
	FVC %	83.7 ± 20.9	83.2 ± 15.2	ns
	FEV1/FVC	52.7 ± 15.9	63.9 ± 13.6	0.01

Ig (mg/dL)	IgA	182 ± 74	237 ± 114	ns
	IgM	309 ± 124	110 ± 53	<0.01
	IgG	812 ± 198	920 ± 310	ns

IgG subclass (mg/dL)	IgG1	472 ± 105	544 ± 226	ns
	IgG2	290 ± 128	302 ± 132	ns
	IgG3	63 ± 30	74 ± 63	ns
	IgG4	19 ± 17	26 ± 26	ns

## References

[1] Anwar GA, McDonnell MJ, Worthy SA (2013). Phenotyping adults with non-cystic fibrosis bronchiectasis: a prospective observational cohort study. *Respiratory Medicine*.

[2] Perry KM, King DS (1940). Bronchiectasis. *American Review of Tuberculosis*.

[3] Simpson JL, Grissell TV, Douwes J, Scott RJ, Boyle MJ, Gibson PG (2007). Innate immune activation in neutrophilic asthma and bronchiectasis. *Thorax*.

[4] Pasteur MC, Helliwell SM, Houghton SJ (2000). An investigation into causative factors in patients with bronchiectasis. *American Journal of Respiratory and Critical Care Medicine*.

[5] Nick JA, Rodman DM (2005). Manifestations of cystic fibrosis diagnosed in adulthood. *Current Opinion in Pulmonary Medicine*.

[6] Casals T, de Gracia J, Gallego M (2004). Bronchiectasis in adult patients: an expression of heterozygosity for CFTR gene mutations?. *Clinical Genetics*.

[7] Miravitlles M, de Gracia J, Rodrigo M-J (1999). Specific antibody response against the 23-valent pneumococcal vaccine in patients with *α*1-antitrypsin deficiency with and without bronchiectasis. *Chest*.

[8] de Gracia J, Rodrigo MJ, Morell F (1996). IgG subclass deficiencies associated with bronchiectasis. *American Journal of Respiratory and Critical Care Medicine*.

[9] Cohen M, Sahn SA (1999). Bronchiectasis in systemic diseases. *Chest*.

[10] Cukier A, Stelmach R, Kavakama JI, Terra Filho M, Vargas F (2001). Persistent asthma in adults: comparison of high resolution computed tomography of the lungs after one year of follow-up. *Revista do Hospital das Clinicas*.

[11] Scala R, Aronne D, Palumbo U (2000). Prevalence, age distribution and aetiology of bronchiectasis: a retrospective study on 144 symptomatic patients. *Monaldi Archives for Chest Disease*.

[12] Dunnill MS (1960). The pathology of asthma, with special reference to changes in the bronchial mucosa. *Journal of Clinical Pathology*.

[13] Paganin F, Séneterre E, Chanez P (1996). Computed tomography of the lungs in asthma: influence of disease severity and etiology. *American Journal of Respiratory and Critical Care Medicine*.

[14] Lynch DA, Newell JD, Tschomper BA, Cink TM, Newman LS, Bethel R (1993). Uncomplicated asthma in adults: comparison of CT appearance of the lungs in asthmatic and healthy subjects. *Radiology*.

[15] Oguzulgen IK, Kervan F, Ozis T, Turktas H (2007). The impact of bronchiectasis in clinical presentation of asthma. *Southern Medical Journal*.

[16] Loftus BG, Price JF, Lobo-Yeo A, Vergani D (1988). IgG subclass deficiency in asthma. *Archives of Disease in Childhood*.

[17] de Moraes Lui C, Oliveira LC, Diogo CL, Kirschfink M, Grumach AS (2002). Immunoglobulin G subclass concentrations and infections in children and adolescents with severe asthma. *Pediatric Allergy and Immunology*.

[18] Domingo Ch (2001). Effectiveness and efficiency of an outpatient clinic for corticosteroid-dependent asthmatics. *Archivos de Bronconeumologia*.

[19] Global Initiative for Asthma (GINA) Global strategy for asthma management and prevention. *NHLBI/WHO Workshop Report*.

[20] Comet R, Domingo Ch, Larrosa M (2006). Benefits of low weekly doses of methotrexate in steroid-dependent asthmatic patients. A double-blind, randomized, placebo-controlled study. *Respiratory Medicine*.

[21] Domingo Ch, Moreno A, Amengual MJ, Comet R, Luján M (2009). Twelve years’ experience with methotrexate for GINA treatment step 5 asthma patients. *Current Medical Research and Opinion*.

[22] Domingo Ch, Moreno A, Amengual MJ, Montón C, Suárez D, Pomares J (2011). Omalizumab in the management of oral corticosteroid-dependent IgE-mediated asthma patients. *Current Medical Research and Opinion*.

[23] Domingo Ch, Moreno A, Mirapeix R (2011). Rationale for the use of immunomodulatory therapies in the Global Initiative for Asthma (GINA) step V asthma other than oral glucocorticosteroids. *Internal Medicine Journal*.

[24] Sanchis J, Casan P, Castillo J (1989). Normativa para la práctica de la espirometría forzada. *Archivos de Bronconeumología*.

[25] Roca J, Sanchis J, Agusti-Vidal A (1986). Spirometric reference values from a Mediterranean population. *Clinical Respiratory Physiology*.

[26] Reiff DB, Wells AU, Carr DH, Cole PJ, Hansell DM (1995). CT findings in bronchiectasis: limited value in distinguishing between idiopathic and specific types. *American Journal of Roentgenology*.

[27] Comet R, Domingo Ch, Navarro M (1998). Bronchiectasis (B) and Immunoglobulin (IG) deficiencies in corticosteroid-dependent asthmatic patients: preliminary results. *Chest*.

[28] de Jong PA, Müller NL, Paré PD, Coxson HO (2005). Computed tomographic imaging of the airways: relationship to structure and function. *European Respiratory Journal*.

[29] Domingo Ch, Roig J (1991). Discinesia ciliar primaria. *Medicina Clínica*.

[30] Domingo Ch, Mirapeix RM, Encabo B, Roig J, López D, Ruiz J (1997). Clínica y ultraestructura de la discinesia biliar primaria y el síndrome de Young. *Revista Clínica Española*.

[31] Domingo Ch, Bosque-García M (2006). Nasal potential difference test to diagnose cystic fibrosis. *Archivos de Bronconeumologia*.

[32] Bisaccioni C, Aun MV, Cajuela E, Kalil J, Agondi RC, Giavina-Bianchi P (2009). Comorbidities in severe asthma: frequency of rhinitis, nasal polyposis, gastroesophageal reflux disease, vocal cord dysfunction and bronchiectasis. *Clinics*.

[33] Pastorino AC, Rimazza RDC, Leone C, Castro APM, Solé D, Jacob CMA (2006). Risk factors for asthma in adolescents in a large urban region of Brazil. *Journal of Asthma*.

